# Supplementary material for: Structural role of essential light chains in the apicomplexan glideosome
Source: Commun Biol. 2020 Oct 13;3:568. doi: 10.1038/s42003-020-01283-8 (PMC7555893; doi:10.1038/s42003-020-01283-8)
Supplement: Supplementary file 1 — Supplementary Information [file 42003_2020_1283_MOESM1_ESM.pdf]

Supplementary Fig. 1

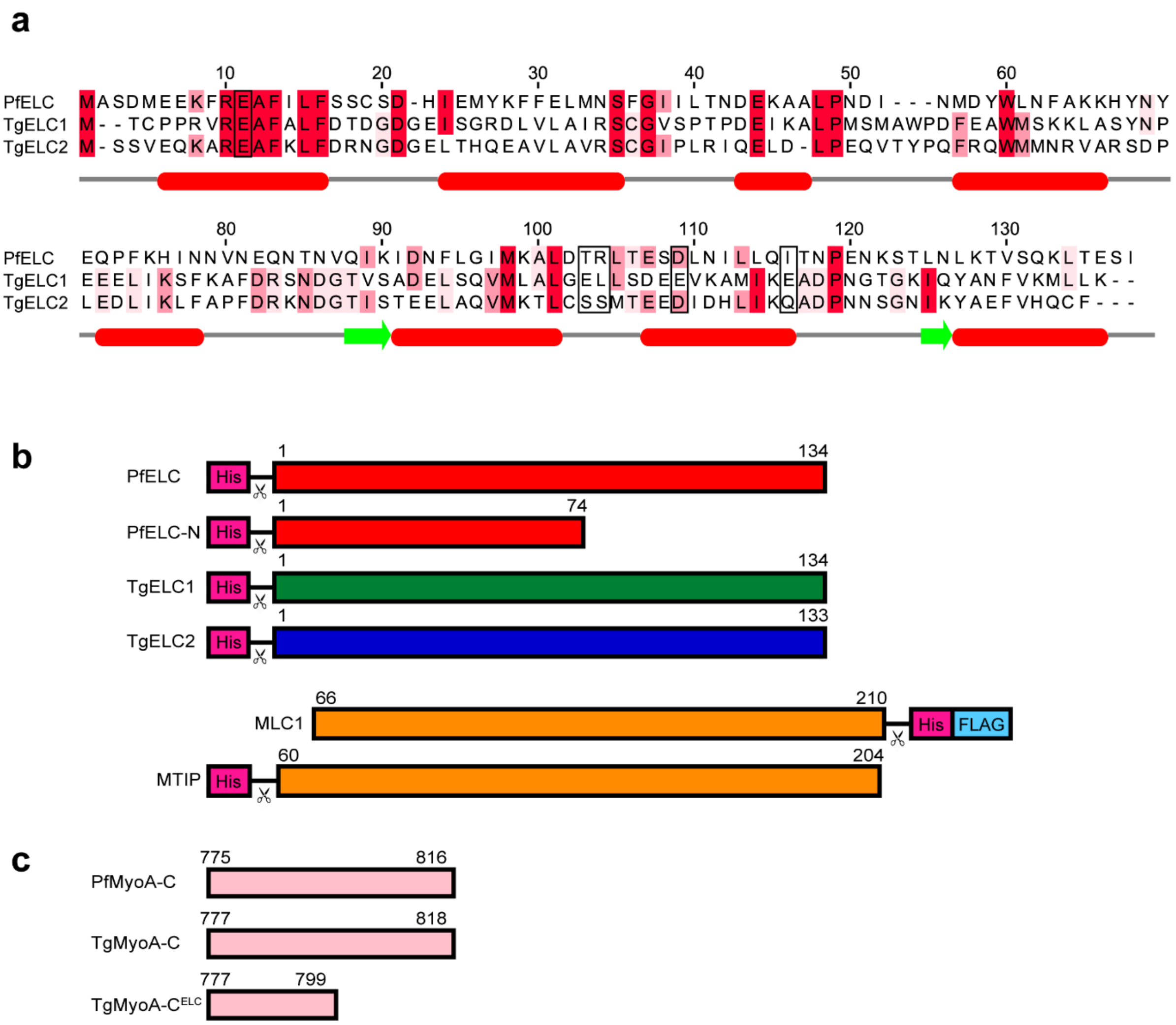

(A) Sequence alignment of *P. falciparum* PfELC and *T. gondii* TgELC1 and TgELC2. Identical residues between these proteins are highlighted in red. The boxed residues indicate the residues involved in the polar interactions with TgMyoA (see Fig. 3c-d and Supplementary Table 6). Secondary structure elements of PfELC as predicted by JPred are graphically shown under the sequence alignment. (B) Schematic representation of the myosin light chain constructs used in this study. The numbers indicate the sequence residues of the particular protein; the scissor symbol represents a TEV cleavage site. (C) The peptide constructs representing the C-terminal regions of MyoAs with indicated domain boarders used in this study. The constructs PfMyoA-C and TgMyoA-C encompass both MLC1/MTIP binding sites as well as the upstream conserved region which binds the essential light chains. The construct TgMyoA-C^{ELC} only consists of the TgELC binding site.

Supplementary Fig. 2

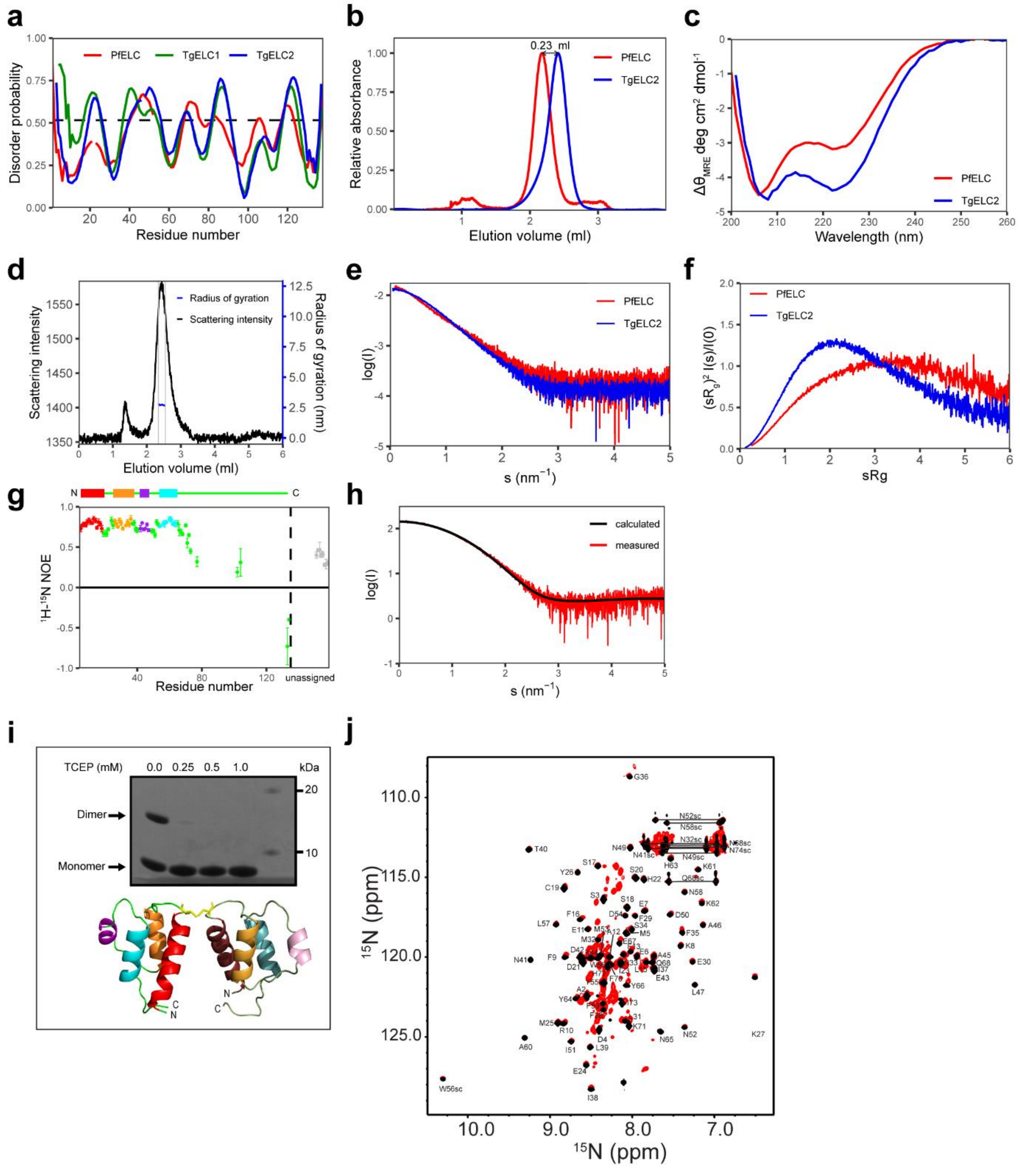

(A) Disorder probability prediction calculated by the disEMBL server shows differences between PfELC and TgELCs, predominantly in the C-terminal region of the sequence. The disorder for the prediction was defined by dictionary of secondary structure probabilities. The amino acid residues with disorder probability above the threshold (dashed line) are predicted to be disordered. (B) Gel filtration profile of PfELC (red) and TgELC2 (blue) on a Superdex

200 5/150 column. PfELC elutes at a smaller elution volume, suggesting that it has a larger hydrodynamic radius compared to TgELC2. (C) Far-UV circular dichroism spectrum of PfELC (red) and TgELC2 (blue) shows that PfELC has a lower α-helical and higher random coil content compared to TgELC2. (D) Elution profile of on-line SEC-SAXS measurement of PfELC using a Superdex 200 5/150 column with the region used for the analysis highlighted in grey. (E) Recorded SAXS curves of isolated PfELC and TgELC2 indicate conformational differences of these homologous proteins. (F) The dimensionless Kratky plot shows that TgELC2 is more compact than PfELC. This is evident because the maximum is considerably closer to $sR_g = \sqrt{3}$ for TgELC2 than PfELC and the plot converges closer to zero in case of TgELC2. (G) Backbone dynamics of PfELC on a picosecond to nanosecond time scale. Heteronuclear NOE ($\{^1H\}$-^{15}N NOE) of PfELC on a residue basis. The C-terminus of PfELC is disordered as indicated by the low heteronuclear NOEs for this region. Residues are colored according to secondary structure elements (four α-helices: from N terminus red, orange, violet, cyan, random coil/loop residues are green, unassigned C-terminal residues in grey). (H) Experimental small angle X-ray scattering curve of PfELC (red) and calculated scattering (black line) from the crystal structure of the PfELC monomer fit with a X^2 value of 1.37, confirming that the protein is a structurally rigid globular monomer in solution. (I) SDS-PAGE gel with PfELC-N samples dialyzed against buffers with varying concentration of TCEP and subsequently alkylated by 2-iodoacetamide and dimer of PfELC-N formed by a cysteine bond between two symmetry related molecules. The results show that the protein is monomeric at the concentration of TCEP used for its biophysical characterization. (J) Overlay of ^{15}N HSQC spectra of full-length PfELC (red) and its N-terminal construct PfELC-N (black), indicating that the construct PfELC-N is identical to the N-terminal domain of full-length PfELC. Assigned resonances are labeled.

Supplementary Fig. 3

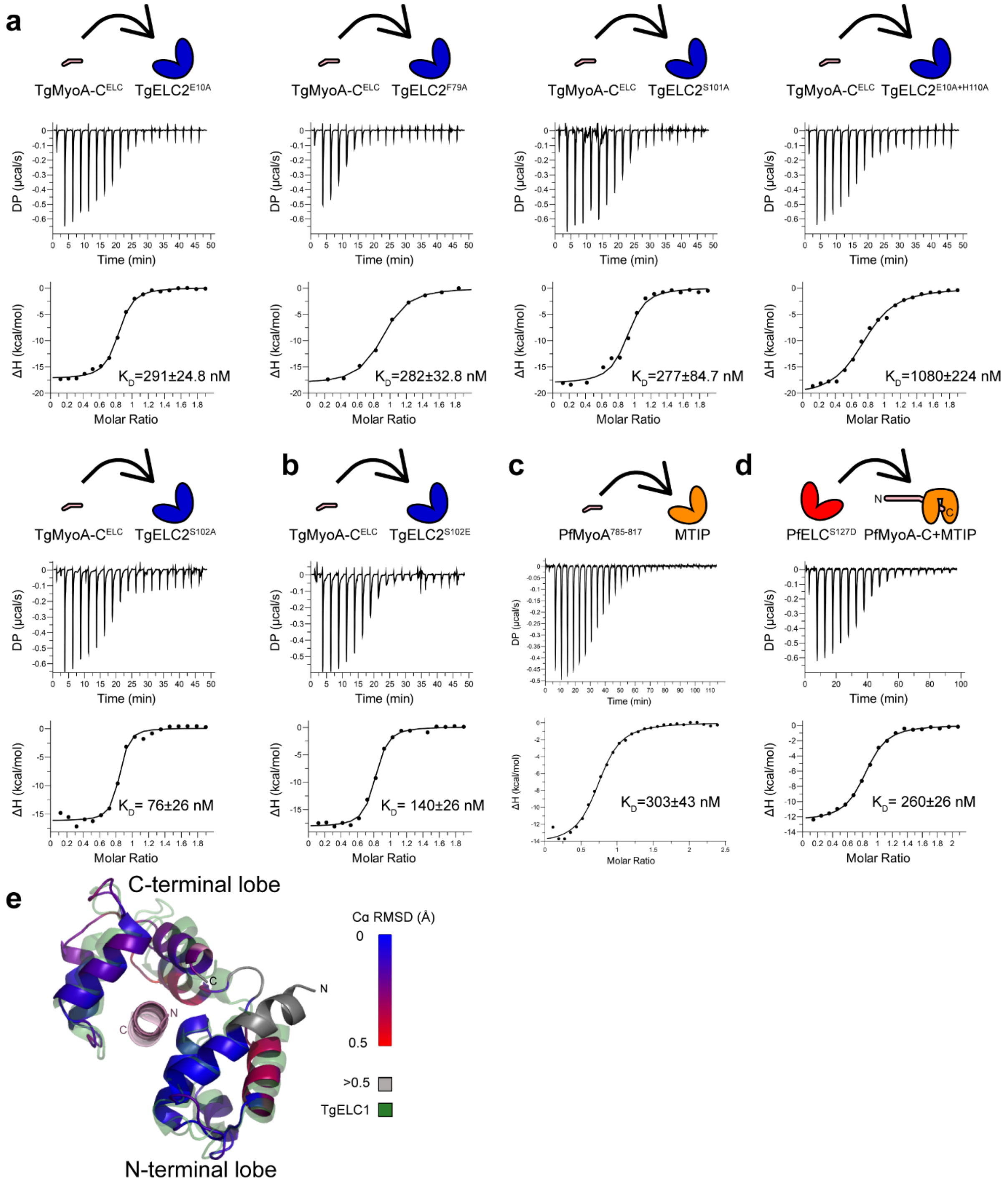

(A) Isothermal titration calorimetry of TgELC2 mutants binding to TgMyoA-C^{ELC}. Individual mutations of polar residues (E10A, F79A, S101A, S102A) of TgELC2 interacting with TgMyoA-C^{ELC} do not cause major changes in the affinity of the two components, but the double mutant TgELC2E10A+H110A shows a substantially lower affinity. (B) Isothermal titration calorimetry of TgELC2 phosphomimetic mutant S102E leads to a small increase of the

resulting K_D which suggests that phosphorylation of S102 is unlikely to regulate complex formation. (C) Isothermal titration calorimetry of PfMyoA$^{785-815}$ titrated into MTIP. (D) Isothermal titration calorimetry of PfELC phosphomimetic mutant S127E causes a twofold decrease in K_D. (E) Structural alignment of TgELC1 and PfELC from the crystal structure of their complexes shows that the C-terminal lobe and helix 4 of PfELC display different conformation than TgELC1.

Supplementary Fig. 4

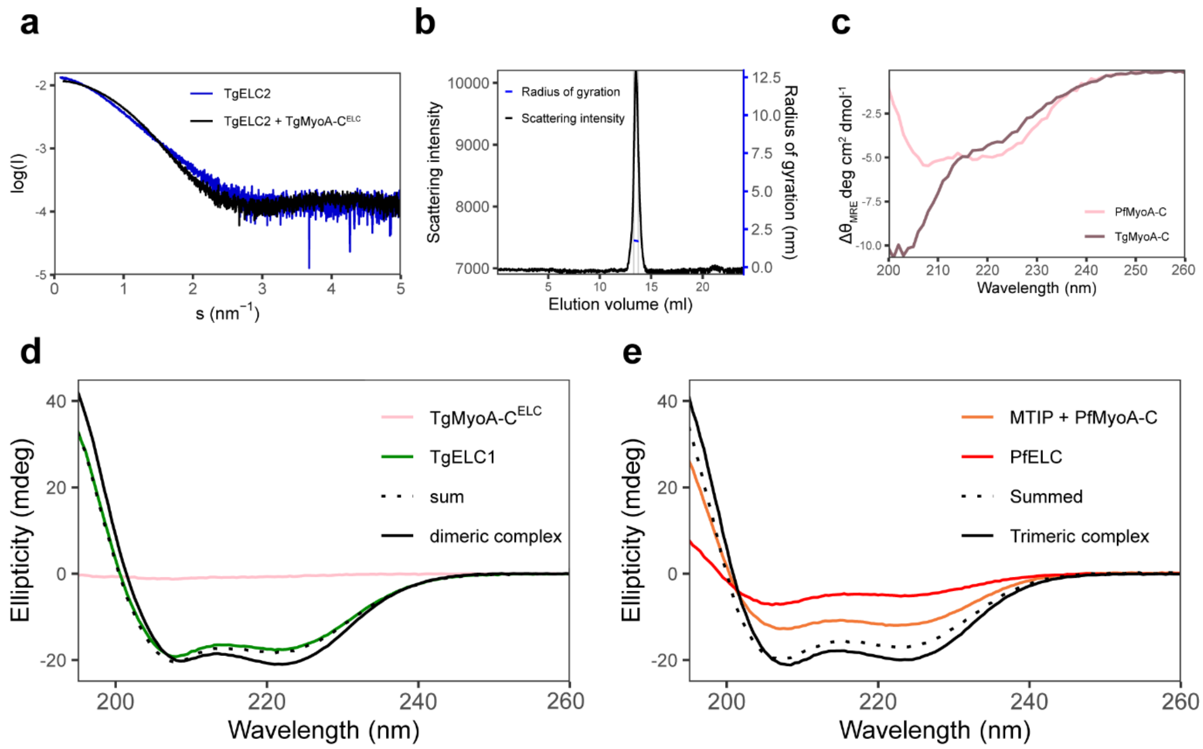

(A) Small Angle X-ray scattering profiles of TgELC2 (blue) and in complex with TgMyoA-C (black) show conformational changes upon interaction. (B) Elution profile of on-line SEC-SAXS measurement of TgELC2 using a Superdex 200 10/300 column with the region used for the analysis highlighted in grey. (C) Far-UV CD spectra of both PfMyoA-C (pink) and TgMyoA-C (violet) indicate that the unbound C-terminus of MyoA is disordered (TgMyoA) or partially disordered (PfMyoA). (D) Far-UV CD data indicate that TgELC1 induces α-helical structure in TgMyoA upon binding. The individual spectra of TgELC1 (green) and TgMyoA^{ELC} (pink) do not sum up (dotted black line) to the spectrum of their dimeric complex (black continuous line), which has more pronounced features of α-helical secondary structure with lower ellipticity at 222 nm and higher ellipticity at 195 nm. (E) The far-UV CD data show that, similarly to TgELCs, the amount of α-helical structure increases upon binding of PfELC to MyoA C-terminus. The data were collected in a 1 mm cuvette at a concentration of 5 μM of each component in 10 mM NaP (pH 7.5), 150 mM NaF and 0.25 mM TCEP at 20°C.

Supplementary Fig. 5

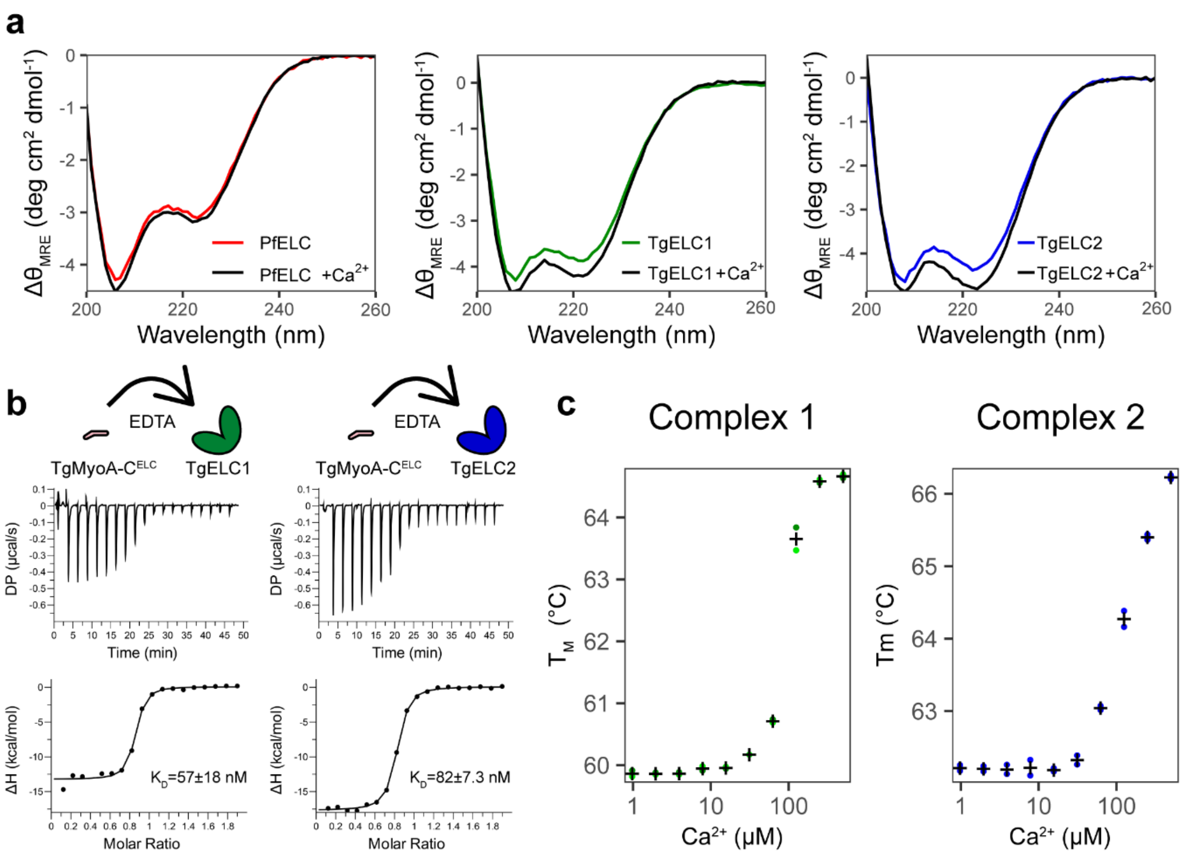

(A) Comparison of far-UV circular dichroism spectra of individual ELC proteins in presence and absence of calcium ions show that calcium does not significantly alter the secondary structure of the ELCs. (B) Binding isotherms of MyoA-C^{ELC} titrated to TgELC1 or TgELC2 in the presence of 5mM EDTA shows that calcium does not have a major influence on the affinity of TgELC2 to the myosin A neck. (C) Stability dependence of the trimeric complex upon addition of increasing concentrations of calcium illustrated by the increase in T_M (°C). Stability data for complex one are shown on the left and for complex two on the right. The colored points are individual measurements and “**+**“ represents the average. The experiment shows that the stability of the trimeric complex is greatly enhanced by the addition of calcium in a concentration-dependent manner.

Supplementary Fig. 6

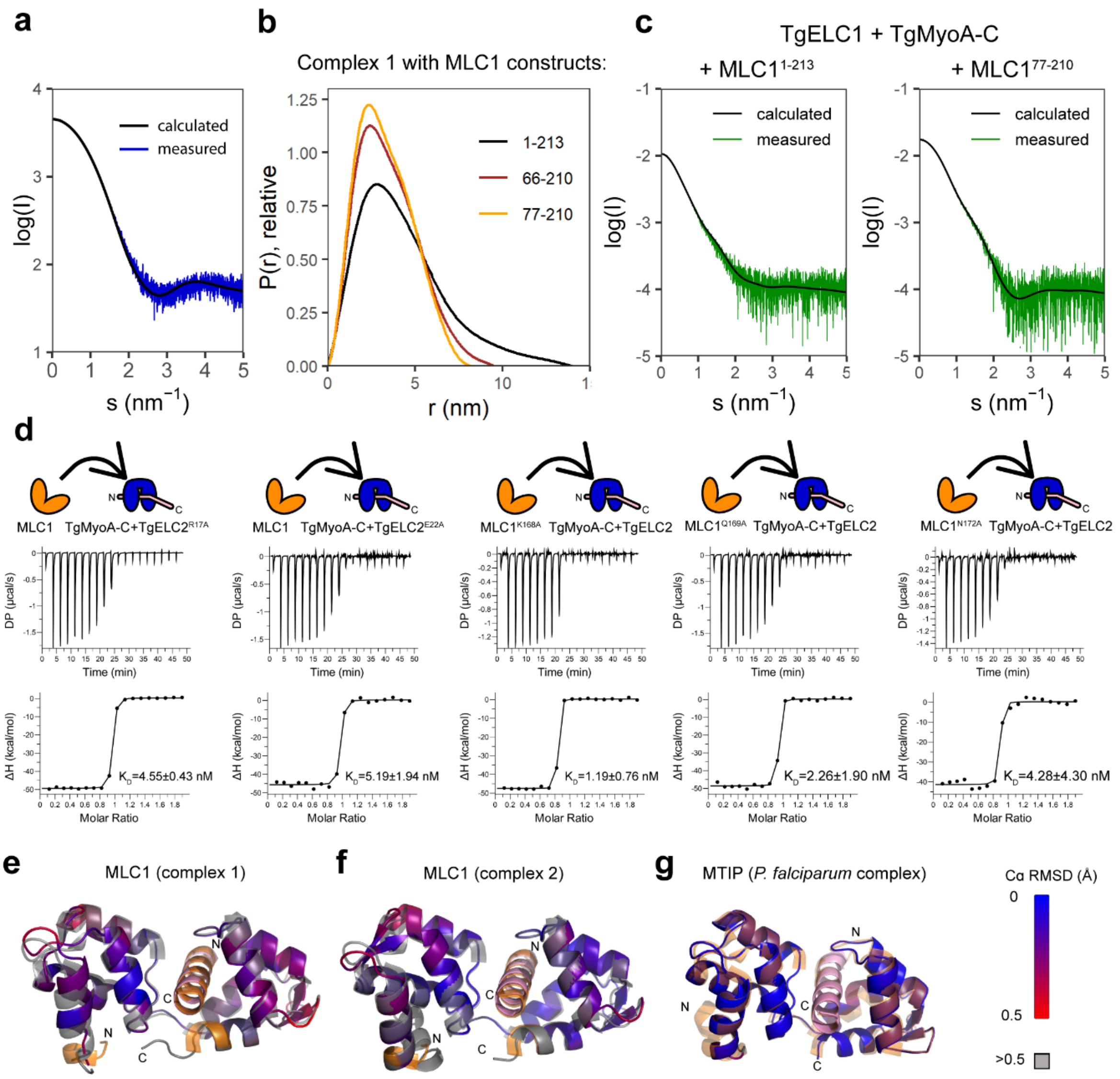

(A) Experimental small angle X-ray scattering curve of TgELC2 bound to TgMyoA-C^{ELC} (blue). The fit to the scattering pattern computed from the crystal structure of complex 2 omitting MLC1 (black line, χ^2 = 1.16) shows that TgELC2 does not undergo major conformational changes upon binding of MLC1 and the formation of the trimeric complex. (B) The distance distribution plots of complex 1 calculated from experimental small angle X-ray scattering data change by shortening the MLC1 N-terminal domain, indicating flexibility of MLC1 upstream of residue 77. The distance distribution is narrower upon N-terminal truncation of MLC1, with d_{max} decreasing from 14 nm (complex 1 with full-length MLC1$^{1-213}$) to 9.5 nm (MLC1$^{66-210}$) and further to 8.2 nm (MLC1$^{77-210}$). (C) Experimental small angle X ray scattering curves of

complex 2 with the short MLC1 construct (MLC1$^{77-210}$) and full-length MLC1 (MLC1$^{1-210}$). The calculated scattering curve computed from the crystal structure of complex 1 fits the scattering data of complex 1 with construct MLC1$^{77-210}$ with $\chi^2 = 1.04$, suggesting that MLC1 residues 77-210 form a folded and rigid entity in the complex. The experimental data of complex 1 with full length MLC1$^{1-213}$ fit the calculated scattering data from the crystal structure of complex 1 and N-terminal MLC1 residues modelled by CORAL with $\chi^2 = 1.15$. (D) ITC binding isotherms of MLC1 titrated to the TgELC2/TgMyoA-C pre-complex. TgELC2 or MLC1 residues forming the binding interface were mutated individually and their affinity was measured to assess the contribution to the binding interface within the trimeric complex. The measured mutants were TgELC2 mutants R17A and E22A, and MLC1 mutants K168A, Q169A and N172A. The binding affinities were in the low nanomolar range. (E-G) Overlays of MLC1 or MTIP derived from the published dimeric complex structure in grey (PDB ID 5vt9 and 4aom, orange) with the protein chains of the trimeric complex structures show that MLC1 and MTIP do not undergo any major structural changes upon ELC binding. The dimeric complex structure agrees with a backbone RMSD of 0.96 Å and 0.75 Å with complex 1 and complex 2, respectively and 1.41 Å with *P. falciparum* complex. The key interactions of TgMyoA with MLC1 are also conserved between the structures of the trimeric and dimeric complexes (R808, H812, R814) with the exception of few weak polar interactions (see Supplementary Table 6). Color code of MLC1 derived from the trimeric complexes according to RMSD deviation of Cα is indicated.

Supplementary Fig. 7

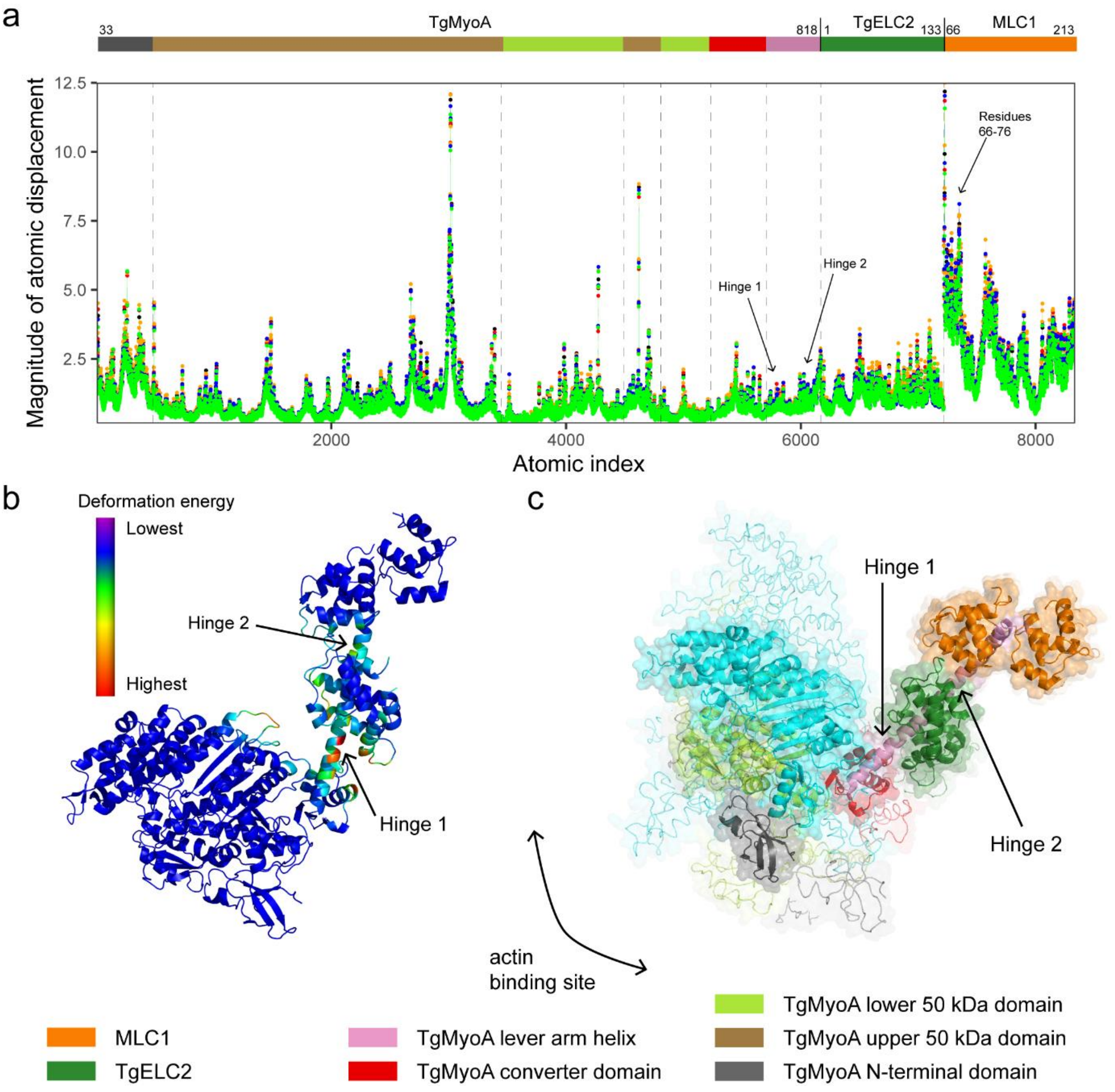

(A) Summary plot of the atomic displacements predicted by NMA based on the five lowest-energy models for complex 2 selected by the lowest clash score. The relative atomic displacement of the individual amino acid residues follows the same pattern in all five models, confirming that the results of the normal mode analysis are independent of the chosen starting conformation or the energy-minimized model. The results for complex 1 are similar (data not shown). (B) The deformation analysis of complex 1 averaged through the 20 lowest-energy modes predicts two main hinge regions, with the hinge 1 (residues 773-777), having the largest contribution to the observed motions. The model is coloured by the deformation energy

from low (violet) to high (red). (C) The ensemble of the structures of complex 2 based on the two lowest-energy modes, which contribute most to the large-scale dynamics of proteins. The original model is drawn in cartoon representation with shown semitransparent surface, whereas the deformed structures are partially transparent and drawn in ribbon representation with faded surface. The structures were aligned on MLC1 to reflect the immobilization of MLC1 in the IMC membrane as in the current model of the glideosome (see Fig. 1a).

Supplementary Table 1. A list of published *P. falciparum* and *T. gondii* glideosome protein structures. So far, only structures of individual proteins of the glideosome and two homologous sub-complexes (MTIP/PfMyoA and MLC1/TgMyoA) have been determined.

**Organism**	**Protein**	**Residues**	**PDB ID**	**Year**	**Ref.**
P. falciparum	GAP50	24-365	3tgh	2012	25
P. falciparum	MyoA	2-768	6i7d, 6i7e	2019	28
T. gondii	MyoA	33-778	6due	2018	27
P. falciparum	MTIP+MyoA	60-204, 799-816	4aom	2012	26
T. gondii	MLC1+MyoA	66-210, 801-831	5vt9	2017	15

Supplementary Table 2. Biophysical characterization and comparison of PfELC and TgELC2.

	SEC data	CD data			SAXS data		
	**Elution volume (ml)**	**α helix**	**β sheet**	**random**	***Rg* (nm)**	**MW (kDa)**	**D_{max} (nm)**
**TgELC2**	2.41	41%	15%	45%	2.15	17.0	6.73
**PfELC**	2.18	34%	16%	51%	2.83	16.3	9.50

Supplementary Table 3. SAXS sample details, data acquisition parameters, structural parameters and atomistic modelling.

**Sample details**									
Sample	**PfELC-N**	**PfELC**	**TgELC2**	**TgELC2 + TgMyoA-C^{ELC}**	**Complex 1 (MLC1$^{1-213}$)**	**Complex 1 (MLC1$^{66-210}$)**	**Complex 1 (MLC1$^{77-210}$)**	**Complex 2 (MLC1$^{66-210}$)**	**Complex of *P. falciparum***
Organism	*P. falciparum*	*P. falciparum*	*T. gondii*	*T. gondii*	*T. gondii*	*T. gondii*	*T. gondii*	*T. gondii*	*P. falciparum*
Source	*E. coli* BL21	*E. coli* BL21	*E. coli* BL21	*E. coli* BL21	*E. coli* BL21	*E. coli* BL21	*E. coli* BL21	*E. coli* BL21	*E. coli* BL21
UniProt ID	Q8IJM4	Q8IJM4	B9PZ33	B9PZ33 + S8G527	XYZ* + S8G527 + Q95UJ7	XYZ* + S8G527 + Q95UJ7	XYZ* + S8G527 + Q95UJ7	B9PZ33 + S8G527 + Q95UJ7	Q8IJM4 + Q8IDR3 + Q8I4Q8
Extinction coefficient ε (at 280 nm, M^{-1} cm^{-1})	11460	8480	16760	25240	49850	35870	35870	30370	35870
Molecular weight from chemical composition (Da)	9011.2	15763.9	15471.5	20681.34	44183.1	37181.1	36619.5	37785.6	37459.5
Concentration (analysis or injection, mg/ml)	3.75	10	5	10	3.9	5.6	4.8	15.5	8.2
Solvent composition	20mM HEPES pH 7.5, 150mM NaCl, 0.5mM TCEP								
**SAS data collection parameters**									
Beamline	P12, DESY/EMBL, Hamburg (Germany)								
Detector	Pilatus 6M								
Energy (kEV)	10.0								
Sample-to-detector distance (mm)	3000								
q-measurement range (Å-^{10})	0.003 – 0.732								
Absolute scaling method	Relative to the scattering of pure water								
Method for monitoring radiation damage	Frame comparison								
Exposed time for frame	1 s (20 x 0.05 s)	900 s (900 x 1 s)	1 s (20 x 0.05 s)	1500 s (1500 x 1 s)	1500 s (1500 x 1 s)	1500 s (1500 x 1 s)	1500 s (1500 x 1 s)	1500 s (1500 x 1 s)	1500 s (1500 x 1 s)
Mode	Batch	SEC-SAXS	Batch	SEC-SAXS	SEC-SAXS	SEC-SAXS	SEC-SAXS	SEC-SAXS	SEC-SAXS
Sample temperature (°C)	20	20	20	20	20	20	20	20	20
**Structural parameters**									
Guinier Analysis									
I(0) (cm^{-1})	0.007±0.001	0.007±0.001	0.013±0.001	0.021±0.001	0.011±0.001	0.029±0.001	0.017±0.001	0.050±0.001	0.037±0.001
R_g (Å)	13.6±1.9	27.2±1.7	21.4±0.5	17.3±0.1	32.4±0.2	26.7±3.0	25.2±4.0	26.7±3.2	27.4±1.8
q-range ($Å^{-1}$)	0.01-0.09	0.01-0.05	0.01-0.06	0.01-0.07	0.01-0.04	0.01-0.05	0.01-0.05	0.01-0.05	0.01-0.05
Fidelity (Quality of fit parameter, *AutoRg*)	0.96	0.93	0.97	0.97	0.93	0.96	0.92	0.98	0.96
Molecular weight (Da)**									
Rel. to standard (BSA, 66 kDa)	9762	n.d.	16824	n.d.	n.d.	n.d.	n.d.	n.d.	n.d.
From V_c	8486	16299	16991	16223	38396	34547	32210	35435	35029
From *MoW*	7311	15636	16700	14536	27615	35069	32610	33378	36809
P(r) analysis									
I(0) (cm^{-1})	0.007±0.001	0.007±0.001	0.013±0.001	0.021±0.001	0.011±0.001	0.029±0.001	0.017±0.001	0.050±0.001	0.038±0.001

R_g (Å)	13.7±0.01	28.3±0.42	21.5±0.05	17.3±0.01	35.0±0.24	27.2±0.05	25.5±0.05	27.4±0.04	28.4±0.05
d_{max} (Å)	43.0	95.0	67.3	54.9	140	95.0	82.0	100	107
q-range (Å^{-1})	0.01-0.59	0.01-0.30	0.01-0.37	0.01-0.46	0.01-0.25	0.01-0.30	0.01-0.32	0.01-0.30	0.01-0.29
Total quality estimate	0.85	0.52	0.80	0.86	0.71	0.84	0.91	0.79	0.78
Porod volume x 10^3 (Å^3)	11.80	22.81	26.72	27.59	63.74	49.68	47.03	49.92	50.76
**Atomistic modelling**	*CRYSOL* with constant subtraction and maximum order of harmonics equal to 50								
Crystal structures	6tj4, chain A, residues 1-68			6tj7, chains A+C (res. 777-798)		6tj6, chains A+B+C	6tj6, chains A+B$^{(res.\ 81\text{-}214)}$+C	6tj7, chains A+B+C	XXX, chains A+B+C
q-range for modelling	0.01-0.50			0.01-0.50		0.01-0.50	0.01-0.50	0.01-0.50	0.01-0.50
χ^2, P-value	1.37, 0.00			1.16, 0.00		1.26, 0.00	1.04, 0.20	2.41, 0.00	3.94, 0.00
Predicted R_g (Å)	13.19			17.31		6.42	25.67	26.26	25.80
Vol (Å), Ra (Å), Dro (e Å^{-3})	10436, 1.760, 0.030			22222, 1.780, 0.025		42336, 1.800, 0.065	46310, 1.400, 0.022	46306, 1.800, 0.050	45540, 1.800, 0.055
	CORAL hybrid rigid body modeling								
Starting crystal structures					6tj6, chains A+B$^{81\text{-}210}$+C				
Flexible residues					1-80 of chain B				
q-range for modelling					0.01-0.58				
χ^2, P-value					1.15, 0.00				
**SASBDB IDs for data and models**									
	SASDH64	SASDH74	SASDH84	SASDH94	SASDHA4	SASDHB4	SASDHC4	SASDHD4	SASDHE4

* the protein with accession number *TGME49_069440* in ToxoDB database has been, as our data show, incorrectly split to two different genes and is not available in the current versions of database.

** calculated from BSA standard for batch mode samples, and V_c and *MoW* volume estimates for SEC-SAXS samples, using the appropriate functions in ATSAS

Supplementary table 4. NMR and refinement statistics for PfELC (residues 1-74).

	Protein
NMR distance and dihedral constraints	
Distance constraints	2320
Total NOE	2320
Intra-residue	900
Inter-residue	1420
Sequential ($\|i-j\| = 1$)	324
Medium-range ($\|i-j\| < 4$)	373
Long-range ($\|i-j\| > 5$)	723
Intermolecular	0
Hydrogen bonds	0
Total dihedral angle restraints	118
ϕ	59
ψ	59
Structure statistics	
Violations (mean and s.d.)	
Distance constraints (Å)	0.03 ± 0.02
Dihedral angle constraints (º)	1.2 ± 0.5
Max. dihedral angle violation (º)	16.5
Max. distance constraint violation (Å)	1.53
Deviations from idealized geometry	
Bond lengths (Å)	0.0023 ± 0.0004
Bond angles (º)	0.38 ± 0.06
Impropers (º)	0.3 ± 0.1
Average pairwise r.m.s. deviation** (Å)	
Heavy	1.23 ± 0.14
Backbone	0.87 ± 0.11

** “Pairwise r.m.s. deviation was calculated among 10 refined structures.”

Supplementary methods

Oligos used for cloning

Plasmodium falciparum MTIP primers for LIC cloning into pNIC28_Bsa4 vector

MTIP_FL_N28_Fw TACTTCCAATCCATGAAACAAGAATGCAATGTATGTTATTTT
MTIP_FL_N28_Rv TATCCACCTTTACTGTTATTGTAATATATCTTCACAGAATAATTTGT

Plasmodium falciparum MTIP-S primers for Slice cloning into pNIC28_Bsa4 vector

MTIPTr_Nt_SliceFW ggtgtagatctgggtaccgagaacctgtacttccaatccatgGAATCAGTTGCTGACATA
MTIPTr_NtSliceRv
tgtcgacggagctcgaattcggatccgtatccacctttactgTTATTGTAATATATCTTCACAGA
A

Plasmodium falciparum ELC primers for Slice cloning into pNIC28_Bsa4 vector

10175_FL_Nt_Fw ggtgtagatctgggtaccgagaacctgtacttccaatccatgATGGCATCTGATATGG
10175_FL_Nt_Rv tgtcgacggagctcgaattcggatccgtatccacctttactgTTATATCGATTCCGTTAA

Plasmodium falciparum ELC-N primers for LIC cloning into pNIC28_Bsa4 vector

pNIC28_ELC_LIC_Fw TACTTCCAATCCATGGCATCTGATATGGAAGAAAAATTTAGAGA
pNIC28_ELC_LIC_N74_Rv
TATCCACCTTTACTGTTAATTAATATGTTTAAATGGTTGTTCATAGTTGTAG
TG

Plasmodium falciparum PfMyoA-C primers for restriction cloning into pETM11_SUMO3 vector

PfMyoA_SUMO_Slice_FW ttccagcaacagaccggtggatccGTTGAATGGGAAAATTGTGTGAGT
PfMyoA_SUMO_Slice_RV gtgctcgagtgcggccgcaagcttTTATACCATTTTTTTTCTTATATGAGC

Toxoplasma gondii TgMyoA-C primers for restriction cloning into pET_GB-1a vector

TgMyoA_pep1_FW
AAAACCATGGCTTCTTCTTGGGAGCCTCTCGTCTCAGTGCTCGAGGCGTA
CTACGCTGGCAGACGCCACAAGAAGCAGCTGC
TgMyoA_pep1_RV
AAAAGGATCCTTACACCAGGTGTCTGCGGATGTGAGCCTGGGCGCGAAT
GATGAAGGGGGTCTTTTTCAGCAGCTGCTTCTTG

Toxoplasma gondii TgMLC1 short constructs primers for LIC cloning into pNIC_CTHF vector

pNIC_CTHF_TgMLC1_66_FW TTAAGAAGGAGATATACTATGGCAGACGAAGACATGCAG
pNIC_CTHF_TgMLC1_77_FW TTAAGAAGGAGATATACTATGGTGGAGGCCGACGAAATG
pNIC_CTHF_TgMLC1_210_RV GATTGGAAGTAGAGGTTCTCTGCCTCGAGCATTGCCTTGC

Plasmodium falciparum PfELC primers to create mutation S127D by blunt end PCR

PfELC_S127D_BEfw TCAAAAATTAACGGAATCGATATAACagta
PfELC_S127D_BErv TCTACTGTCTTAAGGTTTAAGGTTGATTTAT

Toxoplasma gondii TgELC1 primers for extending the purchased gene

ELC1-part1_FW
TACTTCCAATCCATGACCTGCCCGCCGCGTGTTCGTGAAGCGTTCGCGCT
GTTCGACACCGAC
ELC1-part2_RV
CCGCAAGAACGGATCGCCAGAACCAGGTCACGACCAGAGATTTCACCGT
CACCGTCGGTGTCGAACAGCG
ELC1-part3_FW
GCGATCCGTTCTTGCGGTGTTTCTCCGACCCCGGACGAAATCAAAGCGCT
GCCGatgTCAATGGCGTGGCC

Toxoplasma gondii TgELC1 primers for Slice cloning into pNIC28_Bsa4

TgELC1_Slice_FW	ggtgtagatctgggtaccgagaacctgtacttccaatccATGACCTGCCCGCCGCG
TgELC1_Slice_RV	tgtcgacggagctcgaattcggatccgtatccacctttactgTTATTTCAGCAGCATCTTGACAAAG

Toxoplasma gondii TgELC2 primers for single amino acid mutations by blunt-end PCR

TgELC2_E10A_BErv	GCGCGCGCTTTTTGTTCGAC
TgELC2_E10A_BEfw	GGCATTCAAGCTTTTCGATCGC
TgELC2_R17A_BErv	GCATCGAAAAGCTTGAATGCCTCG
TgELC2_R17A_BEfw	GAATGGTGATGGCGAGTTAACG
TgELC2_E22A_BErv	GCGCCATCACCATTGCGA
TgELC2_E22A_BEfw	GTTAACGCATCAAGAAGCTGTCC
TgELC2_F79A_BErv	GCAGGCGCAAACAGTTTGATCAG
TgELC2_F79A_BEfw	GGATCGCAAAAATGATGGCACGA
TgELC2_D80A_BErv	GCGAAAGGCGCAAACAGTTTGATCAG
TgELC2_D80A_BEfw	GCGCAAAAATGATGGCACGATC
TgELC2_G101A_BErv	GCGCAGAGAGTCTTCATGACTTGAGC
TgELC2_G101A_BEfw	GTCTATGACGGAGGAGGACATCG
TgELC2_H110A_BErv	GCATCGATGTCCTCCTCCGTCA
TgELC2_H110A_BEfw	GCTCATTAAACAAGCGGATCCAAACAAC

Toxoplasma gondii MLC1 primers for single amino acid mutations by blunt-end PCR

TgMLC1_K168A_BEfw	GCAGATGGGGAACATCCTCA
TgMLC1_K168A_BErv	GCGCGCGTCAGGTAACCG
TgMLC1_Q169A_BEfw	GATGGGGAACATCCTCATGACC
TgMLC1_Q169A_BErv	GCCTTGCGCGTCAGGTAACCG
TgMLC1_N172A_BEfw	GATCCTCATGACCTACGGAGAGC
TgMLC1_N172A_BErv	GCCCCCATCTGCTTGCGCG

Primers for re-cloning gene cassettes into pPYC vector

NdeI_to_pPYC_FW	AAAAcatatgcaccatcatcatcatcattc
XbaI_to_pPYC_RV	AAAAtctagacgacggagctcgaattcgga
