# Supplementary material for: Structural role of essential light chains in the apicomplexan glideosome
Source: Commun Biol. 2020 Oct 13;3:568. doi: 10.1038/s42003-020-01283-8 (PMC7555893; doi:10.1038/s42003-020-01283-8)
Supplement: Supplementary file 2 — Description of Additional Supplementary Files [file 42003_2020_1283_MOESM2_ESM.pdf]

Description of Additional Supplementary Files

Supplementary data: Interacting residues in the glideosome subcomplexes
