# Supplementary material for: Structural role of essential light chains in the apicomplexan glideosome
Source: Commun Biol. 2020 Oct 13;3:568. doi: 10.1038/s42003-020-01283-8 (PMC7555893; doi:10.1038/s42003-020-01283-8)
Supplement: Supplementary file 3 — Supplementary Data [file 42003_2020_1283_MOESM3_ESM.pdf]

Supplementary Data. Interacting residues in the glideosome subcomplexes.

Complex 1

TgELC1/TgMyoA Interface: 1299.5 (Å²)

Salt bridges

##	TgMyoA	Dist. [Å]	TgELC1
1	C:ARG 793[NH1]	3.96	A:GLU 114[OE1]
2	C:ARG 793[NH2]	2.67	A:GLU 114[OE2]
3	C:ARG 793[NH2]	2.64	A:GLU 114[OE1]
4	C:ARG 794[NE]	3.01	A:GLU 107[OE2]
5	C:ARG 794[NH2]	3.90	A:GLU 107[OE2]
6	C:LYS 796[NZ]	3.10	A:GLU 9[OE1]
7	C:LYS 796[NZ]	3.92	A:GLU 9[OE2]

Hydrogen bonds

1	C:TYR 789[OH]	3.01	A:LEU 132[O]
2	C:TYR 790[OH]	3.20	A:GLU 114[OE1]
3	C:ARG 793[NH2]	2.67	A:GLU 114[OE2]
4	C:ARG 793[NH2]	2.64	A:GLU 114[OE1]
5	C:ARG 794[NE]	3.01	A:GLU 107[OE2]
6	C:ARG 794[NH2]	3.30	A:LEU 102[O]
7	C:GLU 787[OE1]	3.37	A:GLU 101[N]
8	C:GLU 787[OE1]	2.81	A:LEU 102[N]
9	C:GLU 787[OE1]	2.96	A:LEU 103[N]
10	C:GLU 787[OE1]	2.62	A:LEU 103[N]

MLC1/TgMyoA Interface: 1293.3 (Å²)

Salt bridges `

##	TgMyoA	Dist. [Å]	MLC1
1	C:LYS 802[NZ]	3.94	B:GLU 152[OE1]
2	C:ARG 808[NE]	2.70	B:ASP 145[OD1]
3	C:ARG 808[NE]	3.41	B:ASP 145[OD2]
4	C:ARG 808[NH2]	3.59	B:ASP 145[OD1]
5	C:ARG 808[NH2]	2.72	B:ASP 145[OD2]
6	C:HIS 812[ND1]	3.56	B:GLU 212[OE1]
7	C:HIS 812[NE2]	2.59	B:ASP 145[OD2]
8	C:ARG 814[NE]	3.24	B:GLU 185[OE1]
9	C:ARG 814[NE]	2.82	B:GLU 185[OE2]
10	C:ARG 814[NH2]	2.72	B:GLU 185[OE1]
11	C:ARG 814[NH2]	3.76	B:GLU 185[OE2]
12	C:ARG 815[NE]	3.03	B:GLU 212[OE1]
13	C:ARG 815[NH2]	3.90	B:GLU 212[OE1]

Hydrogen bonds

1	C:ARG 808[NE]	2.70	B:ASP 145[OD1]
2	C:ARG 808[NH1]	2.99	B:ALA 111[O]
3	C:ARG 808[NH2]	2.72	B:ASP 145[OD2]
4	C:ARG 808[NH2]	2.93	B:ALA 111[O]
5	C:GLN 810[NE2]	2.92	B:TYR 177[O]
6	C:GLN 810[NE2]	2.90	B:LEU 174[O]
7	C:HIS 812[NE2]	2.59	B:ASP 145[OD2]
8	C:ARG 814[NE]	2.82	B:GLU 185[OE2]
9	C:ARG 814[NE]	3.69	B:TYR 114[OH]
10	C:ARG 814[NH1]	3.01	B:GLU 179[O]
11	C:ARG 814[NH2]	3.08	B:PRO 180[O]
12	C:ARG 814[NH2]	2.72	B:GLU 185[OE1]
13	C:ARG 815[NE]	3.03	B:GLU 212[OE1]
14	C:ARG 815[NH1]	2.93	B:GLU 210[O]
15	C:ARG 815[NH2]	2.65	B:MET 208[O]
16	C:ARG 815[NH2]	2.90	B:GLU 210[O]
17	C:HIS 816[ND1]	2.88	B:TYR 193[OH]
18	C:HIS 816[NE2]	2.70	B:MET 208[O]
19	C:HIS 795[O]	3.75	B:TYR 156[OH]
20	C:GLN 810[OE1]	2.84	B:GLU 179[N]

MLC1/TgELC1 Interface: 316 (Å²)

Salt bridges

##	MLC1	Dist. [Å]	TgELC1
1	B:LYS 168[NZ]	3.60	A:ASP 19[OD1]
2	B:LYS 168[NZ]	3.47	A:GLU 21[OE1]
3	B:LYS 168[NZ]	3.66	A:GLU 21[OE2]

Hydrogen bonds

##	Structure 1	Dist. [Å]	Structure 2
1	B:LYS 168[NZ]	3.60	A:ASP 19[OD1]
2	B:LYS 168[NZ]	3.47	A:GLU 21[OE1]
3	B:LYS 168[NZ]	3.54	A:ASP 19[O]
4	B:GLN 169[NE2]	2.65	A:ASP 17[O]
5	B:ILE 173[N]	3.77	A:GLY 18[O]
6	B:TYR 177[OH]	2.98	A:ALA 12[O]

Complex 2

TgELC2/TgMyoA Interface: 1236.9 (Å²)

Salt bridges

##	TgMyoA	Dist. [Å]	TgELC2
1	C:ARG 794[NE]	3.97	A:ASP 107[OD2]
2	C:ARG 794[NH2]	2.99	A:ASP 107[OD2]
3	C:LYS 796[NZ]	3.13	A:GLU 10[OE1]
4	C:LYS 796[NZ]	2.80	A:GLU 10[OE2]

Hydrogen bonds

1	C:TYR 790[OH]	3.19	A:GLN 114[OE1]
2	C:ARG 793[NH1]	3.61	A:GLN 114[OE1]
3	C:ARG 794[NH2]	2.99	A:ASP 107[OD2]
4	C:LYS 796[NZ]	2.80	A:GLU 10[OE2]
5	C:GLU 787[OE2]	3.04	A:SER 102[N]
6	C:GLU 787[OE2]	2.79	A:SER 102[OG]
7	C:GLU 787[OE2]	3.24	A:SER 101[N]
8	C:TYR 789[OH]	3.61	A:GLN 114[NE2]
9	C:TYR 790[OH]	3.33	A:HIS 110[ND1]

MLC1/TgMyoA Interface: 1305.2 (Å²)

Salt bridges

##	TgMyoA	Dist. [Å]	MLC1
1	C:ARG 808[NE]	3.46	B:ASP 145[OD2]
2	C:ARG 808[NE]	2.98	B:ASP 145[OD1]
3	C:ARG 808[NH2]	2.92	B:ASP 145[OD2]
4	C:ARG 808[NH2]	3.83	B:ASP 145[OD1]
5	C:HIS 812[NE2]	2.75	B:ASP 145[OD2]
6	C:ARG 814[NE]	3.50	B:GLU 185[OE1]
7	C:ARG 814[NE]	2.88	B:GLU 185[OE2]
8	C:ARG 814[NH2]	3.02	B:GLU 185[OE1]
9	C:ARG 814[NH2]	3.80	B:GLU 185[OE2]
10	C:HIS 816[NE2]	3.04	B:GLU 192[OE1]
11	C:HIS 816[NE2]	3.21	B:GLU 192[OE2]

Hydrogen bonds

1	C:ARG 808[NE]	2.98	B:ASP 145[OD1]
2	C:ARG 808[NH1]	2.95	B:ALA 111[O]
3	C:ARG 808[NH2]	2.92	B:ASP 145[OD2]
4	C:ARG 808[NH2]	2.86	B:ALA 111[O]
5	C:GLN 810[NE2]	2.88	B:LEU 174[O]
6	C:GLN 810[NE2]	2.79	B:TYR 177[O]
7	C:HIS 812[NE2]	2.75	B:ASP 145[OD2]
8	C:ARG 814[NE]	2.88	B:GLU 185[OE2]
9	C:ARG 814[NH1]	3.01	B:GLU 179[O]
10	C:ARG 814[NH2]	3.02	B:GLU 185[OE1]
11	C:ARG 814[NH2]	3.11	B:GLU 179[O]
12	C:ARG 814[NH2]	2.75	B:PRO 180[O]
13	C:ARG 815[NH1]	3.70	B:GLU 210[O]
14	C:ARG 815[NH2]	3.47	B:GLU 210[O]
15	C:ARG 815[NH2]	2.79	B:MET 208[O]
16	C:HIS 816[NE2]	3.04	B:GLU 192[OE1]
17	C:GLN 810[OE1]	2.59	B:GLU 179[N]

MLC1/TgELC2 Interface: 403.9 (Å²)

Salt bridges

##	MLC1	Dist. [Å]	TgELC2
1	B:LYS 168[NZ]	3.65	A:GLU 22[OE1]
2	B:LYS 168[NZ]	2.63	A:GLU 22[OE2]

Hydrogen bonds

1	B:TYR 177[OH]	3.18	A:LYS 13[O]
2	B:GLN 169[NE2]	2.84	A:ASN 18[O]
3	B:LYS 168[NZ]	2.63	A:GLU 22[OE2]
4	B:TYR 156[O]	2.83	A:ARG 17[NH1]
5	B:THR 176[O]	3.09	A:LYS 13[NZ]

Complex 1f

TgELC1/TgMyoA Interface: 1257.1 (Å²)

Salt bridges

##	TgMyoA	Dist. [Å]	TgELC1
1	C:ARG 793[NH1]	3.58	A:GLU 114[OE2]
2	C:ARG 793[NH2]	3.30	A:GLU 114[OE1]
3	C:ARG 793[NH2]	3.45	A:GLU 114[OE2]
4	C:ARG 794[NE]	2.98	A:GLU 107[OE2]
5	C:ARG 794[NH2]	3.90	A:GLU 107[OE2]
6	C:LYS 796[NZ]	3.72	A:GLU 9[OE1]
7	C:LYS 796[NZ]	3.06	A:GLU 9[OE2]

Hydrogen bonds

1	C:TYR 789[OH]	3.30	A:LEU 132[O]
2	C:TYR 790[OH]	2.68	A:GLU 114[OE1]
3	C:ARG 793[NH1]	3.58	A:GLU 114[OE2]
4	C:ARG 793[NH2]	3.30	A:GLU 114[OE1]
5	C:ARG 793[NH2]	3.45	A:GLU 114[OE2]
6	C:ARG 794[NE]	2.98	A:GLU 107[OE2]
7	C:ARG 794[NH2]	2.94	A:LEU 102[O]
8	C:LYS 796[NZ]	3.06	A:GLU 9[OE2]
9	C:PRO 781[O]	3.47	A:SER 37[OG]
10	C:GLU 787[OE1]	2.77	A:LEU 102[N]
11	C:GLU 787[OE1]	3.68	A:LEU 103[N]
12	C:GLU 787[OE1]	2.97	A:GLU 101[N]
13	C:TYR 789[OH]	3.58	A:ARG 6[NH2]

MLC1/TgMyoA Interface: 1311.2 (Å²)

Salt bridges

##	TgMyoA	Dist. [Å]	MLC1
1	C:LYS 802[NZ]	2.70	B:GLU 152[OE1]
2	C:LYS 802[NZ]	3.68	B:GLU 152[OE2]
3	C:ARG 808[NE]	3.56	B:ASP 145[OD2]
4	C:ARG 808[NE]	2.86	B:ASP 145[OD1]
5	C:ARG 808[NH2]	2.87	B:ASP 145[OD2]
6	C:ARG 808[NH2]	3.72	B:ASP 145[OD1]
7	C:HIS 812[ND1]	3.33	B:GLU 212[OE1]
8	C:HIS 812[NE2]	2.90	B:ASP 145[OD2]
9	C:ARG 814[NE]	2.63	B:GLU 185[OE2]
10	C:ARG 814[NE]	3.60	B:GLU 185[OE1]
11	C:ARG 814[NH2]	3.59	B:GLU 185[OE2]
12	C:ARG 814[NH2]	3.05	B:GLU 185[OE1]
13	C:ARG 815[NE]	2.95	B:GLU 212[OE1]
14	C:ARG 815[NH2]	3.71	B:GLU 212[OE1]

Hydrogen bonds

1	C:LYS 802[NZ]	2.70	B:GLU 152[OE1]
2	C:ARG 808[NE]	2.86	B:ASP 145[OD1]
3	C:ARG 808[NH1]	2.87	B:ALA 111[O]
4	C:ARG 808[NH2]	2.97	B:ALA 111[O]
5	C:ARG 808[NH2]	2.87	B:ASP 145[OD2]
6	C:GLN 810[NE2]	2.85	B:TYR 177[O]
7	C:GLN 810[NE2]	2.99	B:LEU 174[O]
8	C:HIS 812[NE2]	2.90	B:ASP 145[OD2]
9	C:ARG 814[NE]	2.63	B:GLU 185[OE2]
10	C:ARG 814[NE]	3.49	B:TYR 114[OH]
11	C:ARG 814[NH1]	3.24	B:GLU 179[O]
12	C:ARG 814[NH2]	2.89	B:PRO 180[O]
13	C:ARG 814[NH2]	3.05	B:GLU 185[OE1]
14	C:ARG 815[NE]	2.95	B:GLU 212[OE1]
15	C:ARG 815[NH2]	2.52	B:GLU 210[O]
16	C:ARG 815[NH2]	2.92	B:MET 208[O]
17	C:HIS 816[ND1]	3.14	B:TYR 193[OH]
18	C:HIS 816[NE2]	2.96	B:MET 208[O]
19	C:HIS 795[O]	3.70	B:TYR 156[OH]
20	C:GLN 810[OE1]	2.94	B:GLU 179[N]

MLC1/TgELC1 Interface: 327.4 (Å²)

Salt bridges

##	MLC1	Dist. [Å]	TgELC1
1	B:LYS 168[NZ]	2.77	A:GLU 21[OE2]

Hydrogen bonds

1	B:LYS 168[NZ]	2.77	A:GLU 21[OE2]
2	B:GLN 169[NE2]	2.43	A:ASP 17[O]
3	B:ASN 172[ND2]	3.07	A:GLY 18[O]
4	B:ASN 172[ND2]	3.39	A:ASP 15[OD2]
5	B:ILE 173[N]	3.88	A:GLY 18[O]
6	B:TYR 177[OH]	2.85	A:ALA 12[O]

5vt9

MLC1/TgMyoA

Salt bridges

##	TgMyoA	Dist. [Å]	MLC1
1	C:ARG 808[NE]	3.52	A:ASP 145[OD2]
2	C:ARG 808[NE]	2.89	A:ASP 145[OD1]
3	C:ARG 808[NH1]	2.96	A:ASP 145[OD2]
4	C:ARG 808[NH1]	3.79	A:ASP 145[OD1]
5	C:HIS 812[NE2]	2.61	A:ASP 145[OD2]
6	C:ARG 814[NE]	3.51	A:GLU 185[OE1]
7	C:ARG 814[NE]	2.87	A:GLU 185[OE2]
8	C:ARG 814[NH2]	2.85	A:GLU 185[OE1]
9	C:ARG 814[NH2]	3.73	A:GLU 185[OE2]

Hydrogen bonds

1	C:ARG 808[NE]	2.89	A:ASP 145[OD1]
2	C:ARG 808[NH1]	2.96	A:ASP 145[OD2]
3	C:ARG 808[NH1]	2.79	A:ALA 111[O]
4	C:ARG 808[NH2]	2.87	A:ALA 111[O]
5	C:GLN 810[NE2]	2.82	A:TYR 177[O]
6	C:GLN 810[NE2]	2.96	A:LEU 174[O]
7	C:HIS 812[ND1]	3.48	A:MET 208[O]
8	C:HIS 812[NE2]	2.61	A:ASP 145[OD2]
9	C:ARG 814[NE]	2.87	A:GLU 185[OE2]
10	C:ARG 814[NH1]	2.73	A:GLU 179[O]
11	C:ARG 814[NH2]	2.88	A:PRO 180[O]
12	C:ARG 814[NH2]	2.85	A:GLU 185[OE1]
13	C:ARG 815[NH2]	3.16	A:ARG 106[O]
14	C:HIS 816[NE2]	3.34	A:ALA 207[O]
15	C:HIS 816[NE2]	2.72	A:MET 208[O]
16	C:SER 823[OG]	2.68	A:GLU 185[OE2]
17	C:ALA 830[N]	2.80	A:GLY 178[O]
18	C:GLN 810[OE1]	2.73	A:GLU 179[N]
19	C:PRO 824[O]	2.64	A:TYR 114[OH]

Plasmodium trimeric complex

PfELC/PFMyoA Interface: 1421.2 (Å²)

Salt bridges

##	PfMyoA	Dist. [Å]	PfELC
1	C:LYS 792[NZ]	3.86	A:ASP 105[OD1]
2	C:LYS 794[NZ]	3.86	A:ILE 134[O]
3	C:GLU 785[OE1]	3.16	A:ARG 100[NE]
4	C:GLU 785[OE1]	3.49	A:ARG 100[NH2]

Hydrogen bonds

1	C:SER 774[OG]	2.96	A:GLN 79[OE1]
2	C:LYS 790[NZ]	3.24	A:SER 34[O]
3	C:LYS 792[NZ]	3.86	A:ASP 105[OD1]
4	C:LYS 794[NZ]	3.00	A:SER 133[O]
5	C:LYS 794[NZ]	3.86	A:ILE 134[O]
6	C:GLU 785[OE1]	3.04	A:ARG 100[N]
7	C:TYR 793[OH]	2.41	A:LYS 27[NZ]

MTIP/PfMyoA Interface: 1378.5 (Å²)

Salt bridges

##	PfMyoA	Dist. [Å]	MTIP
1	C:ARG 806[NE]	3.45	B:ASP 139[OD1]
2	C:ARG 806[NE]	2.84	B:ASP 139[OD2]
3	C:ARG 806[NH1]	3.86	B:ASP 139[OD1]
4	C:ARG 806[NH2]	2.91	B:ASP 139[OD2]
5	C:HIS 810[NE2]	3.05	B:ASP 139[OD2]
6	C:ARG 812[NH1]	3.11	B:GLU 179[OE1]
7	C:ARG 812[NH1]	3.53	B:GLU 179[OE2]
8	C:LYS 814[NZ]	2.92	B:ASP 201[OD2]

Hydrogen bonds

1	C:SER 803[OG]	3.07	B:LEU 144[O]
2	C:ARG 806[NE]	2.84	B:ASP 139[OD2]
3	C:ARG 806[NH1]	3.13	B:ALA 105[O]
4	C:ARG 806[NH1]	3.86	B:ASP 110[OD2]
5	C:ARG 806[NH2]	2.91	B:ASP 139[OD2]
6	C:ARG 806[NH2]	2.85	B:ALA 105[O]
7	C:GLN 808[NE2]	3.06	B:LEU 168[O]
8	C:GLN 808[NE2]	2.68	B:TRP 171[O]
9	C:HIS 810[NE2]	3.05	B:ASP 139[OD2]
10	C:ARG 812[NH1]	3.11	B:GLU 179[OE1]
11	C:ARG 812[NH2]	2.62	B:ASP 173[O]
12	C:ARG 812[NH2]	3.42	B:ALA 174[O]
13	C:LYS 813[NZ]	3.36	B:LEU 203[O]
14	C:LYS 814[NZ]	2.92	B:ASP 201[OD2]
15	C:SER 803[OG]	3.59	B:LYS 146[N]
16	C:GLN 808[OE1]	2.72	B:ASP 173[N]

MTIP/PfELC Interface: 327.4 (Å²)

Hydrogen bonds

	MTIP	Dist. [Å]	PfELC
1	B:ASN 153[ND2]	2.89	A:SER 18[O]
2	B:SER 162[OG]	3.63	A:ASP 21[OD1]
3	B:GLN 163[NE2]	2.83	A:CYS 19[O]
4	B:TRP 171[NE1]	2.84	A:GLU 11[OE2]
5	B:ASN 153[OD1]	2.70	A:SER 18[OG]
6	B:GLN 163[O]	3.72	A:CYS 19[SG]
