# Supplementary material for: Structural role of essential light chains in the apicomplexan glideosome
Source: Commun Biol. 2020 Oct 13;3:568. doi: 10.1038/s42003-020-01283-8 (PMC7555893; doi:10.1038/s42003-020-01283-8)
Supplement: Supplementary file 5 — Reporting Summary [file 42003_2020_1283_MOESM5_ESM.pdf]

natureresearch

Corresponding author(s): Christian Loew

Last updated by author(s): Jun 8, 2020

Reporting Summary

Nature Research wishes to improve the reproducibility of the work that we publish. This form provides structure for consistency and transparency in reporting. For further information on Nature Research policies, see Authors & Referees and the Editorial Policy Checklist.

Statistics

For all statistical analyses, confirm that the following items are present in the figure legend, table legend, main text, or Methods section.

n/a	Confirmed	
☐	☒	The exact sample size (*n*) for each experimental group/condition, given as a discrete number and unit of measurement
☐	☒	A statement on whether measurements were taken from distinct samples or whether the same sample was measured repeatedly
☒	☐	The statistical test(s) used AND whether they are one- or two-sided<br>*Only common tests should be described solely by name; describe more complex techniques in the Methods section.*
☒	☐	A description of all covariates tested
☒	☐	A description of any assumptions or corrections, such as tests of normality and adjustment for multiple comparisons
☒	☐	A full description of the statistical parameters including central tendency (e.g. means) or other basic estimates (e.g. regression coefficient) AND variation (e.g. standard deviation) or associated estimates of uncertainty (e.g. confidence intervals)
☒	☐	For null hypothesis testing, the test statistic (e.g. *F*, *t*, *r*) with confidence intervals, effect sizes, degrees of freedom and *P* value noted<br>*Give P values as exact values whenever suitable.*
☒	☐	For Bayesian analysis, information on the choice of priors and Markov chain Monte Carlo settings
☒	☐	For hierarchical and complex designs, identification of the appropriate level for tests and full reporting of outcomes
☒	☐	Estimates of effect sizes (e.g. Cohen's *d*, Pearson's *r*), indicating how they were calculated

Our web collection on statistics for biologists contains articles on many of the points above.

Software and code

Policy information about availability of computer code

Data collection	The diffraction data were collected using MXCuBE v 2x. The NMR data were collected using TopSpin (version 2.1). The SAXS data were recorded using BECQUEREL (version 2.0). The ITC data were recorded using MicroCal PEAQ-ITC Control Software (version 1.1.0.1262). The CD data were recorded using Chirascan Spectrometer Control Panel Application (version 4.7.0.194). The nanoDSF data were recorded using PR.ThermControl (version 2.1.2). The analytical size exclusion chromatography was performed using OpenLab CDS Software (Rev.C.01.06[61]).
Data analysis	The raw diffraction images were processed by XDS (version Mar15, 2019). The data was scaled by aimless (version 0.7.4). The molecular replacement and structure refinement were performed in Phenix (version 1.17.1.3660). The structures were analyzed using PyMol (version 2.0.7), the angle between helices was measured by script anglebetweenhelies.py available on www.pymolwiki.org. The interfaces between the molecules and interacting residues were analysed by PDBe PISA (version v1.52). NMR data were processed with NMRPipe (version linux212_64) and analysed with NMRView (version 9.1). The NMR structure calculations were performed using ARIA (version 2.3). The SAXS data were analysed by programs of program suite ATSAS (version 2.8.4). The ITC data were analysed using MicroCal PEAQ-ITC Analysis Software (version 1.10.1262). The CD data were analysed using DichroWeb, available on http://dichroweb.cryst.bbk.ac.uk/html/home.shtml. The sequences were aligned using MAFFT (version v7.419) and represented in Jalview (version 2.105). Modelling was performed using Modeller (versioin 9.18), R/3.5.1 and PyMol (pymol/2.3.0-foss-2017b-Python-2.7.14). The deformation analysis and normal mode analysis were performed using Bio3D (version 2.3-4).

For manuscripts utilizing custom algorithms or software that are central to the research but not yet described in published literature, software must be made available to editors/reviewers. We strongly encourage code deposition in a community repository (e.g. GitHub). See the Nature Research guidelines for submitting code & software for further information.

Data

Policy information about availability of data

All manuscripts must include a data availability statement. This statement should provide the following information, where applicable:

- Accession codes, unique identifiers, or web links for publicly available datasets
- A list of figures that have associated raw data
- A description of any restrictions on data availability

The datasets generated during and/or analyzed during the current study are available from the corresponding author on request. The data source data underlying the charts in the main and supplementary figures is deposited in Figshare repository under DOI 10.6084/m9.figshare.12769841. Coordinates and structure factors as well as NMR structures were deposited in the PDB at the Research Collaboratory for Structural Bioinformatics (RCSB) with the following identifying codes: 6tj3, 6tj4, 6j5, 6tj6, 6tj7, 6zn3. The averaged and subtracted SAXS data were deposited in SASBDB with the following identifying codes: SASDH64, SASDH74, SASDH84, SASDH94, SASDHA4, SASDHB4, SASDHC4, SASDHD4 and SASDHE4. The structural models of full lengths MyoA-MLC1-ELCs have been uploaded to Zenodo (https://zenodo.org).

Field-specific reporting

Please select the one below that is the best fit for your research. If you are not sure, read the appropriate sections before making your selection.

☒ Life sciences ☐ Behavioural & social sciences ☐ Ecological, evolutionary & environmental sciences

For a reference copy of the document with all sections, see nature.com/documents/nr-reporting-summary-flat.pdf

Life sciences study design

All studies must disclose on these points even when the disclosure is negative.

Sample size	n.a.
Data exclusions	n.a.
Replication	n.a.
Randomization	n.a.
Blinding	n.a.

Reporting for specific materials, systems and methods

We require information from authors about some types of materials, experimental systems and methods used in many studies. Here, indicate whether each material, system or method listed is relevant to your study. If you are not sure if a list item applies to your research, read the appropriate section before selecting a response.

Materials & experimental systems

n/a	Involved in the study
☒	☐ Antibodies
☒	☐ Eukaryotic cell lines
☒	☐ Palaeontology
☒	☐ Animals and other organisms
☒	☐ Human research participants
☒	☐ Clinical data

Methods

n/a	Involved in the study
☒	☐ ChIP-seq
☒	☐ Flow cytometry
☒	☐ MRI-based neuroimaging
